# 

**Published:** 1918-04-06

**Authors:** 


Rates of Subscription: Twelve months, 6/6; Six months, 4/-; three months, 2/-, post free to any address in the United Kingdom. Abroad, Twelve months, 8/8; Six months, 5/-; Three months, 2/6, post free. THE HOSPITAL "Index" to Volume LXII, April 1917 to September 1917, is now ready, price 2½d. per copy, post free. Address The Manager, The Hospital, "The Hospital" Building, 28/29 Southampton Street, Strand, London, W.C. 2.

### Recent Official Publications.

Seventy-ninth Annual Report of the Registrar-General of Births, Deaths, and Marriages in England and Wales. Post free, 5s. 7d.

National Health Insurance Act, 1918. Post free, 8d.

National Health Insurance Act, 1918: Summary of Principal Provisions. Post free, 3d.

Alcohol: Its Action on the Human Organism. Post free, 2s. 10d.

Select Committee on National Expenditure: Report and Proceedings. Post free, 1s.

Select Committee on National Expenditure: First Report (of Session 1918). Post free, 3d.

Quarterly Return and Annual Summary of Marriages, Births, and Deaths Registered in England and Wales. Post free, 1s. 2d.

Local Government Board. Notification of Infectious Diseases (Non-Metropolitan). Post free, 1s.

Local Government Board. Notification of Infectious Diseases (Metropolitan). Post free, 1s.

Maternity and Child Welfare Bill. Post free, 1½d.

(The above may be obtained from H.M. Stationery Office, Imperial House, Kingsway, W.C.)

